# Priorities for quality of life after traumatic brain injury

**DOI:** 10.1371/journal.pone.0306524

**Published:** 2024-07-05

**Authors:** Jasleen Grewal, Kix Citton, Geoff Sing, Janelle Breese Biagioni, Julia Schmidt

**Affiliations:** 1 Rehabilitation Science Graduate Program, University of British Columbia, Vancouver, Canada; 2 Rehabilitation Research Program, Centre for Aging SMART at Vancouver Coastal Health, Vancouver, Canada; 3 Nanaimo Brain Injury Society, Nanaimo, Canada; 4 British Columbia Brain Injury Association, Vancouver, Canada; 5 The Cridge Centre for the Family, Victoria, Canada; 6 CGB Centre for Traumatic Life Losses, Victoria, Canada; 7 Department of Occupational Science and Occupational Therapy, University of British Columbia, Vancouver, Canada; University of Sharjah College of Health Sciences, UNITED ARAB EMIRATES

## Abstract

**Background:**

After traumatic brain injury (TBI), individuals can experience changes to quality of life (QOL). Despite understanding the factors that impact QOL after TBI, there is limited patient-oriented research to understand the subjective priorities for QOL after TBI. This study aims to understand the priorities for QOL after TBI using a group consensus building method.

**Methods:**

The Technique for Research of Information by Animation of a Group of Experts (TRIAGE) method was used to determine priorities for QOL after TBI. In phase one, expert participants were consulted to understand the context of QOL after TBI. In phase two, participants with TBI completed a questionnaire to broadly determine the factors that contributed to their QOL. In phase three, a portion of participants from phase two engaged in focus groups to identify the most relevant priorities. Data was analyzed thematically. In phase four, expert participants were consulted to finalize the priorities.

**Results:**

Phase one included three expert participants who outlined the complexity and importance of QOL after TBI. Phase two included 34 participants with TBI who described broad priorities for QOL including social support, employment, and accessible environments. Phase three included 13 participants with TBI who identified seven priorities for QOL: ensuring basic needs are met, participating in everyday life, trusting a circle of care, being seen and accepted, finding meaning in relationships, giving back and advocating, and finding purpose and value. In phase four, four expert participants confirmed the QOL priorities.

**Interpretations:**

Findings emphasize the critical need to address priorities for QOL after TBI to ensure improved health outcomes.

## Introduction

Traumatic brain injury (TBI) is the leading cause of death and disability worldwide, with approximately 50 million individuals impacted globally per year [1]. Individuals with moderate to severe TBI experience high rates of poor health outcomes including physical [2,3] and cognitive disability [4,5]. Additionally, individuals with TBI can experience longstanding health and social consequences such as depression [6], homelessness [7], difficulty returning to work or maintaining employment [8] and changes in interpersonal relationships [9]. These consequences can significantly impact an individual’s ability to resume everyday life. Notably, most of these individuals experience negative changes in quality of life (QOL) after the TBI [10,11].

QOL after TBI is a complex and broad concept, defined as a subjective measure of an individual’s position within a cultural context, which incorporates their values, goals, expectations, function and concerns [12]. QOL includes health related QOL and subjective QOL. Health related QOL includes health domains such as physical, emotional, and social functioning; these aspects affect an individual’s body structure and functions [13]. Subjective QOL can include life satisfaction, self-esteem, and subjective well-being and can be impacted by individual goals (e.g., expectations and priorities) and achievements (e.g., performance, status, health) [14]. Individuals with TBI experience negative changes to both health related and subjective QOL [15].

QOL is a multi-dimensional construct and can be impacted by biological, psychological and social elements. The biopsychosocial model can help describe the influences on QOL by examining the holistic and interconnected nature of biological, psychological and social factors [16]. Biological factors include bodily health and disease, psychological factors include mental and emotional wellness and behaviour, and social factors include interpersonal factors such as community activities and social interactions. The biopsychosocial model emphasizes the interconnectedness of these factors when experiencing health or disease [16]. QOL is a holistic construct and the determinants of QOL can be understood using the biopsychosocial model as biological, psychological and social factors independently and interdependently impact an individual’s subjective experience of QOL. Past research has determined many factors that contribute to QOL [17].

Despite the growing understanding of the complexities of impacts on QOL after TBI, there has been limited research that is co-developed by individuals with TBI to understand their priorities for QOL. Understanding the factors that impact QOL and priorities for improving QOL from the perspective of individuals who have sustained a TBI is key to promoting programs and interventions to improve QOL after TBI. Thus, the aim of this study is to understand the priorities for QOL after TBI using a group consensus building process.

## Methods

### Study design

**Fig 1:**
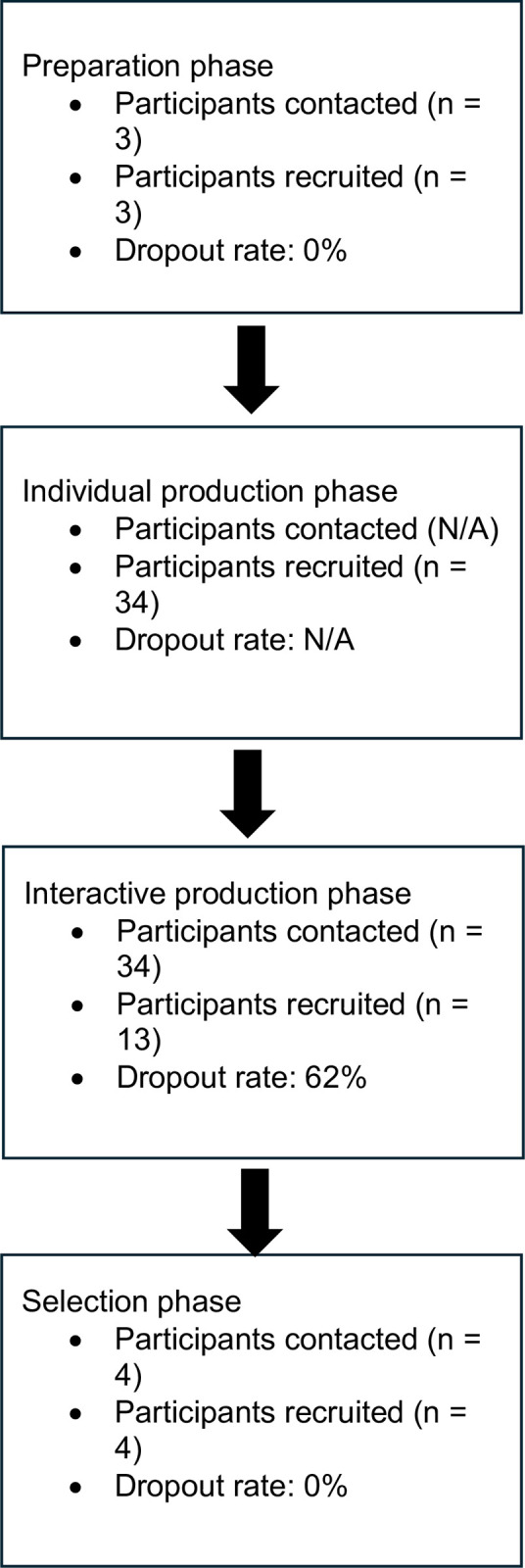
Study flowchart.

The Technique for Research of Information by Animation of a Group of Experts (TRIAGE) [18] method was used as an inductive and structured method to obtain group consensus. There are four phases of the TRIAGE method: (1) preparation; (2) individual production; (3) interactive production (4) selection [18]. In phase one (preparation), a group of expert participants developed the definitions of key concepts and evaluation questions. In phase two (individual production), an online questionnaire was sent to participants with TBI to determine factors that impact QOL. In phase three (interactive production), participants from the previous stage met in virtual focus groups to identify, by consensus, the most relevant priorities among those listed previously. In phase four (selection) the most relevant indicators from the previous stage were finalized and prioritized by expert participants in a virtual meeting. See Fig 1 for study flowchart. Ethics approval was obtained by the Research Ethics Board of the University of British Columbia (H21-03968).

### Procedure

Convenience sampling was used to recruit expert participants for phase one and four and participants with TBI for phase two and three from British Columbia, Canada. Advertisements were circulated within GF Strong Rehabilitation Centre, BC Brain Injury Association, and other community organizations. Inclusion criteria for participants with TBI were (1) individuals aged between 18 to 64 years, (2) self-reported diagnosis of a moderate to severe TBI and (3) able to communicate in English. Individuals who sustained a mild TBI were excluded from the study as their rehabilitation pathways and recovery trajectories differ from moderate to severe TBI. Inclusion criteria for expert participants were individuals with lived experience, caregivers or involved with TBI service delivery (e.g., clinician, policy-maker, community organization leader, etc.). Participants provided written and oral consent prior to data collection.

#### Preparation phase (phase one)

The preparation phase lasted 3-months (August 2021 –October 2021). JS sent emails to contacts at community organizations to recruit expert participants (e.g., individuals with lived experience, caregivers, leaders of community organizations) to define and contextualize QOL after TBI. Expert participants provided oral consent prior to participating, documented in a secured file and witnessed by the lead author (JG). Experts were contacted by e-mail to request participation in an online meeting (via Zoom) and details were provided regarding determinants of QOL after TBI. The meeting was facilitated by researchers JG and AC, recorded (audio and video) and transcribed verbatim by a research assistant. Using this data, we created a questionnaire for phase two.

#### Individual production phase (phase two)

The individual consultation phase lasted 11-months (November 2021 –September 2022) with data collection occurring in the last 6-months. To ensure a breadth of responses and confirm our findings, we recruited additional participants for this phase from August 2023 to October 2023. During this phase, participants with TBI were recruited via in-person posters at GF Strong Rehabilitation Centre and through community organizations. Participants were prompted to email JG if they were interested to participate. Then, an online Qualtrics questionnaire link was sent via email. Community organization leaders also sent the Qualtrics link via email to individuals who matched the inclusion criteria. Participants provided written consent prior to participation. The questionnaire consisted of 17 questions about demographics as well as, semi-structured questionnaires asking about their priorities for QOL after TBI. For the semi-structured questions, participants could input as many factors that impact their QOL. Participants received $20.00 CAD honorarium for participation via electronic transfer. The data was summarized in tables using the Person-Environment-Occupation model [19]. Using this data, we created a focus group guide for phase three.

#### Interactive production phase (phase three)

The interactive production phase lasted 7-months (October 2022 –April 2023) with data collection occurring in the first 3-months. To ensure a breadth of responses and confirm our findings, we recruited additional participants for this phase from November 2023 to December 2023. Participants with TBI from phase two were contacted via e-mail to determine interest for participation in a 1-hour online (via Zoom) focus group. Participants provided oral consent before participating, documented in a secured file and witnessed by the lead author (JG). Three focus groups were conducted with a total of 13 participants, moderated by two research assistants who received training in conducting interviews with individuals with TBI. Focus group participants were informed of the interview aims and moderators’ backgrounds. Questions and discussions focused on QOL after TBI and life experiences, including priorities for building and enhancing QOL (S1 Appendix). Participants received $30.00 CAD honorarium for participation via electronic transfer. No participants withdrew from the study and there were no prior relationships between participants and researchers. The focus groups were recorded (audio and video) and transcribed verbatim by a research assistant. In phase four, we asked expert participants to rate the relevance of data from phase three.

#### Selection phase (phase four)

The selection phase was conducted in April 2023 and was moderated by researcher JG. During this phase, four expert participants (two from the phase one) were recruited and provided with the results from phase three via Qualtrics. Experts provided oral consent prior to participating, documented in a secured file and witnessed by the lead author (JG). Experts then reflected individually if they agreed or disagreed with the listed priorities for QOL after TBI and provided feedback. Experts then collectively, through consensus, determined whether the priorities for QOL after TBI aligned with their personal and professional experience and knowledge. Experts received a $20.00 CAD honorarium for participation via electronic transfer.

### Data analysis

The transcribed meeting from the preparation phase was reviewed by researchers JG and AC. Key points from the meeting were summarized in a Microsoft Word document. Data from phase two was summarized in tables on Microsoft Excel using the Person-Environment-Occupation model [19]. The focus group transcripts from phase three were inductively analyzed following Braun and Clarke’s (2006) thematic analysis approach [20]. During the analysis, JG and JS completed a detailed read of all transcripts to become familiar to the data. To remain close to the data and capture the different priorities for QOL after TBI, JG conducted comprehensive line-by-line coding of transcripts using Microsoft Excel. After initial coding, JG and JS reviewed and discussed the codes. JG and JS collaboratively generated initial priorities through review of codes and transcripts. Then, JG and JS reviewed the initial priorities and collaboratively refined and named them. To ensure trustworthiness, the researchers (JG and JS) employed reflexivity, involved multiple investigators during data analysis and ensured participant verification of findings. Researchers met to discuss their perspectives on the codes and their own biases. There were multiple researchers involved during data analysis to acknowledge the different perspectives on the participants’ data. The completion of phase four allowed experts in TBI to rate the relevance of the priorities.

## Results

### Preparation phase (phase one)

Three expert participants were recruited including two people with lived experience (E1, E2) and a caregiver and leader of a community organization (E3). All participants contacted participated in phase one. We recruited expert participants with different roles to ensure a breadth of experiences and opinions were shared. They reported that the main priorities for QOL after TBI included familial support, receiving healthcare services, and engaging in meaningful activities (e.g., school and employment).

### Individual production phase (phase two)

Thirty-four participants with TBI completed the survey to understand their QOL priorities. Many participants did not disclose their biological sex and gender (n = 18). Of the 16 that responded, 14 were cis-gender, with 7 identifying as male and man and 7 identifying as female and woman. Two participants identified as male sex and woman gender and female sex and man gender. Participants were aged between 25 to 64 years, with 23 participants being above 50 years. Participants had a range of employment positions, many were receiving disability assistance (n = 9) or employed part-time (n = 10), see Table 1.

**Table 1:** Participant demographics (phase two).

	Age (years)	Time since injury (years)	Biological sex and gender	Employment status
**P1**	50 to 54	More than 20	Not reported	Part time employment
**P2**	55 to 59	More than 20	Not reported	Part time employment
**P3**	60 to 64	Less than 5	Not reported	Retired
**P4**	50 to 54	More than 20	Not reported	Part time employment
**P5**	35 to 39	5 to 9	Not reported	Retired
**P6**	60 to 64	More than 20	Not reported	Full time employment
**P7**	60 to 64	5 to 9	Not reported	Retired
**P8**	55 to 59	More than 20	Not reported	Unemployed
**P9**	55 to 59	5 to 9	Not reported	Unemployed
**P10**	45 to 49	More than 20	Not reported	Retired
**P11**	30 to 34	10 to 14	Not reported	Part time employment
**P12**	55 to 59	More than 20	Not reported	Part time employment
**P13**	55 to 59	More than 20	Not reported	Full time employment
**P14**	45 to 49	15 to 19	Not reported	Disability assistance
**P15**	55 to 59	10 to 14	Not reported	Part time employment
**P16**	55 to 59	10 to 14	Not reported	Part time employment
**P17**	55 to 59	1 to 4	Not reported	Disability assistance
**P18**	55 to 59	5 to 9	Not reported	Disability assistance
**P19**	50 to 54	10 to 14	Male and Man	Full time employment
**P20**	45 to 49	More than 20	Female and Woman	Unemployed
**P21**	60 to 64	10 to 14	Female and Woman	Disability assistance
**P22**	50 to 54	15 to 19	Male and Man	Disability assistance
**P23**	60 to 64	10 to 14	Male and Man	Disability assistance
**P24**	40 to 44	10 to 14	Female and Woman	Part time employment
**P25**	30 to 34	10 to 14	Female and Woman	Retired
**P26**	55 to 9	10 to 14	Female and Woman	Unemployed
**P27**	60 to 64	More than 20	Male and Man	Disability assistance
**P28**	35 to 39	More than 20	Male and Woman	Part time employment
**P29**	60 to 64	10 to 14	Male and Man	Retired
**P30**	45 to 49	1 to 4	Female and Woman	Unemployed
**P31**	60 to 64	10 to 14	Female and Woman	Disability assistance
**P32**	50 to 54	10 to 14	Male and Man	Part time employment
**P33**	25 to 30	5 to 9	Female and Man	Unemployed
**P34**	45 to 49	More than 20	Male and Man	Disability assistance

Results from this phase indicated that the main factors that influence QOL included personal factors (e.g., social support and physical health), environmental factors (e.g., accessible healthcare and transportation) and activity-related factors (e.g., engaging in employment) (S1 Table). Participants also determined the most relevant factors that impacted their QOL (Table 2). Participants reported that the main factors that hindered QOL were barriers to social participation, poor physical health and cognition (Table 3). Participants also indicated details regarding the support they were receiving and if they felt well supported after TBI (S2 Table).

**Table 2:** Most relevant influencers of QOL (phase two).

Most Relevant Influencers of QOL	Number of respondents
**Person factors**	
Social (e.g., relationships, support, roles, interactions)	21
Physical health	9
Recreation	8
Overall health	6
Well-balaned diet	5
Financial capacity	5
Cognition	3
Sleep	4
Housing	3
Spirituality	2
Ability to perform daily tasks	2
Mental health	2
Taking medication	1
Managing symptoms of TBI	1
Self-acceptance	1
**Activity-related factors**	
Employment capacity	1
**Environment factors**	
Accessible healthcare	3
Accessible transportation	1
Access to informational resources	1

**Table 3:** Factors that hinder QOL (phase two).

Factors that hinder QOL	Number of respondents
**Person factors**	
Physical health concerns	19
Social barriers	6
Cognitive concerns	7
Reduced sleep	3
Reduced sense of self	2
Overall health concerns	1
Mental health concerns	3
Difficulty obtaining medication	1
Unbalanced diet	2
Substance use	1
Difficulty with self-acceptance	1
Inability to perform daily tasks	3
**Activity-related factors**	
Lack of finances	8
Barriers to employment	2
Increased expenses	1
**Environment factors**	
Overstimulating environment	3
Not feeling understood or accepted by community, stigma	5
Lack of healthcare services	3
Unstable housing	1
Covid-19 pandemic	1

### Interactive production phase (phase three)

All participants from phase two were contacted to participate in phase three. Thirteen participants from the phase three participated in four focus groups to discuss priorities of QOL after TBI (Table 4). Of the participants, six were cis-gender, two identified as male sex and woman gender and female sex and man gender and five did not report their biological sex and gender. Participants were aged between 25 to 64 years with seven participants being over 50 years. Seven priorities were generated based on data obtained from the focus groups (Table 5).

**Table 4:** Participant demographics (phase three).

	Age (years)	Time since injury (years)	Biological sex and gender	Employment status
**P5**	35 to 39	5 to 9	Not reported	Retired
**P9**	55 to 59	5 to 9	Not reported	Unemployed
**P14**	45 to 49	15 to 19	Not reported	Disability assistance
**P15**	55 to 59	10 to 14	Not reported	Part time employment
**P17**	55 to 59	1 to 4	Not reported	Disability assistance
**P21**	60 to 64	10 to 14	Female and Woman	Disability assistance
**P22**	50 to 54	15 to 19	Male and Man	Disability assistance
**P24**	40 to 44	10 to 14	Female and Woman	Part time employment
**P27**	60 to 64	More than 20	Male and Man	Disability assistance
**P28**	35 to 39	More than 20	Male and Woman	Part time employment
**P32**	50 to 54	10 to 14	Male and Man	Part time employment
**P33**	25 to 30	5 to 9	Female and Man	Unemployed
**P34**	45 to 49	More than 20	Male and Man	Disability assistance

**Table 5:** Finalized priorities for QOL after TBI.

Priority	Description
Ensuring basic needs are met	Experiencing financial security, housing, having support for daily tasks
Participating in everyday life	Physical access to the environment through assistive technology, living independently and completing daily activities
Trusting a circle of care	Support from TBI support groups, relationships with individuals who understand TBI, connecting with individuals who care
Being seen and accepted	Self-accepting TBI, being accepted by others, feeling understood and known, feeling valued
Finding meaning in relationships	Impactful interactions with family and friends, engaging socially
Giving back and advocating	Giving back to self, society and peers through advocating, volunteering in the community, sharing lived experiences of TBI, challenging stigma from TBI
Achieving purpose and value	Finding purpose in daily life, determination to achieve goals, interest and motivation to pursue new activities and skills

#### Priority 1: Ensuring basic needs are met

Participants emphasized the importance of having their fundamental needs met to improve QOL. Participants reported housing and financial security were important factors of QOL. For example, one participant described the importance of having enough finances for comfortable living:

“I think finances were the biggest challenge for us, from going from a relatively comfortable living to all of a sudden having to, you know, literally getting in line on welfare day to get your money from a social service worker…” (P14).

A participant described that having a safe space to live predicted their QOL however, they stated that they may not be able to afford this space which impacted how supported they felt, “…I’m very grateful that I’m here in a clean, safe place, that I can, right now, afford, but that’s going to be ending here pretty soon too. So I yeah, I don’t feel supported at all…” (P15). Participants also expressed that support for daily activities was necessary for their QOL. A participant who lived in supportive housing described, “I now live in a group home with 24-hours of support with five brain injury survivors” (P22). However, participants described access to services may change depending on where they reside, “being injured young and in a place outside of Canada, it is difficult to obtain services as you have to pay for these services” (P28).

#### Priority 2: Participating in everyday life

Participants indicated that engaging and participating in daily life was a priority for improving their QOL. Some participants noted the importance of a mobility aid to access the environment, noting “I do have balance issues. So my sticks help a lot” (P15). Similarly, participants described that access to technology, although it can be expensive and inaccessible, has improved their QOL through ability to participate in activities.

Another participant explained how the environment can limit participation and QOL, describing the barriers in the environment, “I can’t imagine having to go back into the workforce, like for me it would just feel like chaos. With noise, constant movement of people, the constant talking, probably music in the background…” (P9). Participants described the importance of participating in productive activities to support QOL after TBI including going back to school and being employed. Participants also described how increasing their independence in daily activities improved their QOL:

“So right now I would say that my quality of life is… I’m very happy with what I’m able to do…from someone, like I said, who was curled up in a ball to now being able to stand. I can have a conversation, go to the bank, withdraw money…So now I’m becoming more and more independent for myself, more confident as well and more socially integrated with mankind” (P22).

Participants reflected that when they tried to return to productive occupations, they became more cognizant of their changed abilities after TBI, noting “when my medical EI ran out, I went back to work, and that was when it really became clear. Oh, I’m not who I used to be. I’ve got real challenges” (P33). Additionally, a participant described that achieving independence after TBI has been a long journey but they are slowly regaining their independence, “So I was in a long-term care facility at first and now I’ve been moved to independent living where I have my own apartment. And yeah, it’s been a long road, but we’re getting there” (P5).

#### Priority 3: Trusting a circle of care

Participants emphasized the importance of having individuals to trust who can provide support. Particularly, one participant described the difficulty and negative impact on QOL when trying to access support from healthcare professionals, “It took like a year to see a psychiatrist to get medication worked out, but once everything was worked out, my quality of my life got so much better…” (P14). Further, participants reflected they often felt guilt when they received support from healthcare professionals, especially if they did not look visibly injured:

“So I got very fortunate, very lucky that this doctor at [hospital] actually took me in… I don’t feel awkward going in to talk to him…because he knows how I feel. I tell him straight up, you know, I appreciate everything you’ve done for me but sometimes I feel guilty for coming in here and seeing what other people have gone through” (P32).

Other participants described being connected to TBI support groups helped them connect to peers, receive support, and engage in exercise. For example, one participant noted:

“So things that I’ve used in the community that have been very valuable to me, it’s called the [group name]. Through that I developed relationships and friendships with persons who could really relate and empathize with what I was going through and what my needs were” (P24).

However, participants also expressed that some groups can be unhelpful, noting “I was in a support group for a little while, which was good, but a little bit triggering as well, so it’s kind of a mixed bag. It helps, but then it kind of retriggers things” (P17). Participants reported finding individuals who listen and care, and having a trusting circle of care to be quite important in the rehabilitation journey, “The biggest thing is to try and find people who will also listen when you’re trying to deal with everything in your life that has changed, right?” (P21). Participants noted that if they could not find others who understand, they experienced loneliness.

#### Priority 4: Being seen and accepted

Participants emphasized the importance of their injury being acknowledged and accepted by others, and self-accepting the TBI and the changes that come with having a TBI. For example, a participant stated he is grateful for the support he has received after TBI, “Accept yourself. I would like to just end off by saying yes, I’m a grateful brain injury survivor who’s got nothing but the best support for the last 17-years” (P22). However, participants indicated that self-acceptance was initially difficult, as they considered pursuing activities from their pre-injury life such as work, “I think I wanted to deny that I had a brain injury or that I couldn’t work through it and become normal again” (P21). Similarly, participants described that it was difficult to acknowledge that their skills and capacity have changed after TBI. Participant described that although their abilities and activities have changed, they experience self-acceptance and that “[It will] take me a little bit more than it would take for somebody else to…. That’s okay. That’s just where I’m at. I’m on the right path” (P9). Participants described how TBI can often be an invisible injury, describing that they often did not feel seen or accepted:

“People don’t, they just don’t get it. We look fine, you know like even getting on… getting on the bus. I absolutely cannot stand on the bus. I absolutely can’t. And do you think anybody like any kids will move out of the handicapped spots? No, I have to ask the bus driver like I absolutely have to sit down…” (P9).

Additionally, a participant described that if others had trouble accepting their injury, it resulted in them feeling like they were not normal,

“Because eventually, you develop this thought that because you have a brain injury, that you’re not normal. So that was how it was implanted in my mind. So when I moved here, to Canada, it took me a while to have self acceptance towards it… I’ll accept it. I do have a brain injury” (P28).

The same participant described that even if others did not accept their injury, it did not mean their injury did not exist, stating “I have to be like, more open now towards like, hey, I am not normal. I have a brain injury. If you can’t accept that, that’s fine with me but you know the truth so that’s all” (P28). Participants described that they feel value when they engage in activities or with individuals who see them for more than their injury, “I think what really brings me value is engaging in activities or engaging with persons where I’m seen for more than the injury, when I’m seen for who I am and not just labeled as the injury” (P24).

#### Priority 5: Finding meaning in relationships

Participants described the importance of having purposeful relationships with friends, family, pets, and strangers and engaging in social activities. Participants emphasized that having friends and family to support during all stages of recovery was meaningful. For example:

“One relationship that comes to mind and I feel really, really grateful for is my best friend. She knew me before the injury and she has stood by my side during and after, and that means a lot. She’s wonderful. She always listens to me and, you know, she empathizes with me. She helps me strategize, and she provides a lot of emotional support and a lot of fun. So I think, like a friend who has stood by you through all the stages of recovery is really, really meaningful” (P24).

A participant described that their service dog was helpful in social situations and encouraged communication with others:

“People want to talk to me about my dogs. They don’t notice my brain injury. It’s not something I have to volunteer. It’s like… there’s something to talk about and so it takes the heat off of me. So it’s nice” (P34).

However, participants described that their relationships changed after TBI and highlighted the barriers to having meaningful relationships such as, finances, noting “…a dramatic change in finances after injury can affect your ability to socialize as you once knew it” (P24). Another barrier was the capacity to be social, “You don’t know what’s going on the inside and I would start to try and see friends for lunch or coffee and then have to cancel because I wasn’t feeling well” (P17).

#### Priority 6: Giving back and advocating

Participants described that supporting the society by advocating, volunteering and peer support, was a priority for QOL. For example, participants described engaging with survivors of TBI instilled purpose and drive, “Everyone’s a survivor and one professor to give their perspective…And this is gives me pleasure. Gives me purpose, gives me drive” (P22). A participant described that volunteering within community organizations and being a peer-support worker improves QOL after TBI:

“That’s what keeps me going on a daily basis is doing that work and volunteering with the [community organization] and that has been my saving grace. It really has. So I know that if I can continue my little part time job working with the kids and volunteering. That’s the best thing that I can do, and it’s definitely giving me my quality of life back….But deep down, I’m trying to make myself happy and in the process hopefully make other people happy” (P15).

Participants described that helping others in similar situations allowed them to reflect on their recovery journey, “And it goes both ways where it’s, for me, being able to reflect on how far I’ve come and also provide support to someone who’s just beginning their journey” (P33).

Participants also highlighted the importance of self-advocacy such as, being on their own and having to advocate for themselves, “You’re left on your own, and that was a real challenge for me…learning that I need to be my own advocate…” (P14). Other participants described that self-advocacy was needed however, it was often difficult, “I find we have to be our own advocate and that is often really difficult to do. You know, it’s hard to know who to reach out to… So yeah, often it’s difficult to be our own advocate” (P34). Participants described developing creative solutions, such scheduling activities during the day to ensure they could engage in social activities with their changed capacity. Participants also emphasized the difficult process of challenging stigmatizing thoughts related to TBI in settings such as, the workplace, “Through my employment, I have experienced a lot of oppression and discrimination and bullying in regards to injury…And I always try to challenge those thoughts, but it’s hard that there’s stigma out there” (P24). The participant also stated that it would be ideal if there could be education and awareness to reduce the stigma and discrimination stemming from TBI.

#### Priority 7: Achieving purpose and value

Participants described how they found purpose in their daily life after TBI and emphasized the interest, motivation, and determination to pursue new skills and goals. A participant stated they learned new skills after TBI, “I have developed a skill set that I believe will only come with lived experience that has been incredibly valuable in the role that I occupy. I do believe I’ve developed great patience and great empathy skills” (P24).

A participant highlighted his experience of finding activities that are stimulating and motivating, noting “…to get out and find other areas that you can get connected to somehow, something that stimulates you, something that motivates you, some way of expressing yourself” (P27). Lastly, a participant described the drive, purpose and motivation with their recovery, even 17-years after injury, “Here we are 17-years post-accident and I continue to get support to this day with my recovery because I keep making small but noticeable progress with my recovery. I have drive, I have purpose, I have determination” (P22).

#### Selection phase (phase four)

Four expert participants (two from phase one) completed the survey to finalize the priorities for QOL after TBI. All participants contacted participated in phase four. The experts included a person with lived experience (E2), a caregiver and leader of a community organization (E3) and two leaders of community organizations (E4, E5). All experts rated the priorities from phase three as highly or moderately relevant (S3 Table). When asked to provide overall feedback for the findings, an expert participant emphasized that individuals, their injury and needs vary, and they need access to support throughout their lives:

“Every person is different, and every brain injury is different. What a survivor needs today may be needed for the remainder of their life. Or what they need today is temporary and it also can be sporadic throughout life. Whatever it is, they need access to those supports when they need it…” (E5).

## Discussion

The current study identified the priorities for QOL from individuals with lived experience of TBI. To our knowledge, this is the first study to identify the priorities using a patient-partnered inductive and structured consensus building approach with individuals with TBI. In this discussion, we will discuss each priority, relevant research, and implications as well as, outline the limitations and future directions.

Findings indicated that meeting basic needs are a priority for QOL. Indeed, research suggests that individuals with TBI can experience unstable housing [7], limited access to healthcare services [21], decreased financial stability due in part to loss of income [22,23], and reduced participation in pre-injury roles [24,25]. Studies on transitional living supports such as housing and policy-related funding can improve participation and financial stability [26] as well as, long-term independent living [10], which are factors known to influence QOL [27,28]. Additionally, research has also identified areas of need post-discharge for indiciduals with TBI including the need for emotional support, instrumental support (e.g., assistance with housekeeping), professional support and community support [27,29,30]. This highlights the importance of the provision of basic needs after TBI.

Participants indicated they prioritized participation in everyday life. Research shows that individuals who were more physically independent and able to complete daily living independently (e.g., showering, dressing), experienced improved QOL [27]. Participation in life after TBI is complex and impacted by many factors; for example, a person may have reduced physical and cognitive capacity to engage in active recreation and therefore, have fewer social connections [31]. A qualitative research study with individuals with brain injury showed that participants felt they participated in daily life if they made choices and decisions about the activities they engaged in, were provided support to engage in activities, could help others, and engage in activities they could tolerate [32]. Importantly, there was a process of re-evaluating current activities that they found meaningful after injury and a process of prioritizing activities that helped them feel good [32]. As such, improving participation in everyday activities and roles is critical to prioritize in rehabilitation.

Further, findings indicated that individuals with TBI benefited from a trusting circle of care and meaningful relationships to facilitate improved QOL. Previous research emphasizes that receiving social support such as, being married, getting along with individuals, available emotional support and being satisfied with the support is positively related to QOL [27,33]. However, after TBI, individuals often experience changes in their relationships [34] and reduced social support [27]. Research has shown that social connections and support are essential for recovery particularly, relationships and social support can help individuals engage in their daily activities [24,35]. Participation in daily activities is also an essential component of QOL [27]. This highlights a critical need for individuals to engage in social activities and receive necessary support after TBI to ensure improved QOL.

Being accepted and valued was identified as a priority for QOL. Social identity theory describes that individuals can develop a sense of identity from social group memberships [36]. Being part of a social group is important for adjustment during periods of adverse life transitions as social groups can provide individuals with resources (e.g., social, psychological, and material) to cope and develop a sense of self [37–40]. Research also shows that becoming a part of a social group after TBI can result in growth such as, having new priorities, increased engagement with others, greater appreciation for life and an inner strength to overcome difficulties [41]. While there are benefits from receiving support and being part of a social group after TBI, previous research indicates that individuals may experience less support after their injury with less satisfaction with the support they are receiving [42]. This highlights a critical need to address these priorities for QOL in research.

Another priority for QOL that aligns with previous research was contributing to society through volunteer work or advocating. Advocacy for self is an important part of managing life after TBI [43]. Self-advocacy relates to self-determination which is needed when making choices and decisions to direct one’s life [44,45] Further, research has shown that contributing to society through volunteering, and sharing experiences with peers and researchers can improve psychological adjustment, and result in higher life satisfaction and a renewed sense of self after TBI [46–48]. Previous studies have shown that when individuals with TBI reflect on their life, they can recognize needs in other individuals with disabilities, therefore experiencing empathy for others [24,49]. Indeed, experiencing empathy is an important area of development after TBI; individuals who experience reduced empathy after TBI can experience negative impacts to their social roles, which could further reduce QOL [50,51]. This highlights a need for clinicians and leaders of community organizations to build capacity for individuals with TBI to contribute to their community to improve empathy and further develop connections after TBI.

Finding purpose and value was identified as a priority for QOL. Given the significant change in function and participation after TBI, individuals may experience a shift in their daily life [24]. This “reshaped reality” may require a person to navigate and restart their new life after injury [24,52]. Research indicates that embracing new challenges, developing strategies, accessing supports, and finding activities that match their reshaped reality may facilitate QOL after injury [24,52]. Interventions such as developing resilience, discovering abilities to redefine oneself and focusing on values or beliefs regarding reasons for living can help self-awareness and self-identity after brain injury [52].

Our study had three main limitations. First, participants were recruited through brain injury organizations in British Columbia and therefore may have received support (e.g., social and physical assistance). As such, participants may not be representative of the general population of individuals with TBI, which may limit the generalizability of our findings. However, participants who were connected to brain injury organizations may give context to the priorities for QOL from the perspective of individuals who are well supported and they may be reflecting on their recovery journey when they were less supported. This can also provide insight to the priorities for QOL of individuals who are less supported. Next, participants were only included if they could complete an online questionnaire and participate in a 60-minute focus group, which excludes individuals with limited technological capacity and cognitive or communication challenges. However, participants in our sample all experienced moderate to severe TBIs, with many of them experiencing sufficient recovery to participate. As such, these participants were able to reflect on the early stages after injury when their impairments created greater participation challenges. Last, we did not make it mandatory for participants to indicate their sex, gender, or culture and did not ask how this impacts QOL in our focus groups. More research is needed to understand how sex, gender and culture influences QOL.

## Conclusion

Our study described seven distinct priorities to improve QOL by individuals with lived experiences of TBI. Research and policy can address these priorities to ensure integration into clinical rehabilitation. There is a critical need to address negative changes to QOL after TBI to ensure improved health outcomes and participation in everyday life.

## Supporting information

S1 Appendix Focus group discussion guide. (DOCX)

S1 Table Factors that influence QOL (phase two). (DOCX)

S2 Table Support after TBI (phase two). (DOCX)

S3 Table Relevance of priorities. (DOCX)
