# Correction: Priorities for quality of life after traumatic brain injury

**DOI:** 10.1371/journal.pone.0328391

**Published:** 2025-07-15

**Authors:** Jasleen Grewal, Kix Citton, Geoff Sing, Janelle Breese Biagioni, Julia Schmidt

There is an error in Tables 1 and 4. The information listed under “Biological sex and gender” for P28 is incorrect. The correct information is “Female and Woman.” Please see the correct Tables 1 and 4 here.

**Table 1:** Participant demographics (phase two).

	Age (years)	Time since injury (years)	Biological sex and gender	Employment status
**P1**	50–54	More than 20	Not reported	Part time employment
**P2**	55–59	More than 20	Not reported	Part time employment
**P3**	60–64	Less than 5	Not reported	Retired
**P4**	50–54	More than 20	Not reported	Part time employment
**P5**	35–39	5–9	Not reported	Retired
**P6**	60–64	More than 20	Not reported	Full time employment
**P7**	60–64	5–9	Not reported	Retired
**P8**	55–59	More than 20	Not reported	Unemployed
**P9**	55–59	5–9	Not reported	Unemployed
**P10**	45–49	More than 20	Not reported	Retired
**P11**	30–34	10–14	Not reported	Part time employment
**P12**	55–59	More than 20	Not reported	Part time employment
**P13**	55–59	More than 20	Not reported	Full time employment
**P14**	45–49	15–19	Not reported	Disability assistance
**P15**	55–59	10–14	Not reported	Part time employment
**P16**	55–59	10–14	Not reported	Part time employment
**P17**	55–59	1–4	Not reported	Disability assistance
**P18**	55–59	5–9	Not reported	Disability assistance
**P19**	50–54	10–14	Male and Man	Full time employment
**P20**	45–49	More than 20	Female and Woman	Unemployed
**P21**	60–64	10–14	Female and Woman	Disability assistance
**P22**	50–54	15–19	Male and Man	Disability assistance
**P23**	60–64	10–14	Male and Man	Disability assistance
**P24**	40–44	10–14	Female and Woman	Part time employment
**P25**	30–34	10–14	Female and Woman	Retired
**P26**	55–9	10–14	Female and Woman	Unemployed
**P27**	60–64	More than 20	Male and Man	Disability assistance
**P28**	35–39	More than 20	Female and Woman	Part time employment
**P29**	60–64	10–14	Male and Man	Retired
**P30**	45–49	1–4	Female and Woman	Unemployed
**P31**	60–64	10–14	Female and Woman	Disability assistance
**P32**	50–54	10–14	Male and Man	Part time employment
**P33**	25–30	5–9	Female and Man	Unemployed
**P34**	45–49	More than 20	Male and Man	Disability assistance

**Table 4:** Participant demographics (phase three).

	Age (years)	Time since injury (years)	Biological sex and gender	Employment status
**P5**	35–39	5–9	Not reported	Retired
**P9**	55–59	5–9	Not reported	Unemployed
**P14**	45–49	15–19	Not reported	Disability assistance
**P15**	55–59	10–14	Not reported	Part time employment
**P17**	55–59	1–4	Not reported	Disability assistance
**P21**	60–64	10–14	Female and Woman	Disability assistance
**P22**	50–54	15–19	Male and Man	Disability assistance
**P24**	40–44	10–14	Female and Woman	Part time employment
**P27**	60–64	More than 20	Male and Man	Disability assistance
**P28**	35–39	More than 20	Female and Woman	Part time employment
**P32**	50–54	10–14	Male and Man	Part time employment
**P33**	25–30	5–9	Female and Man	Unemployed
**P34**	45–49	More than 20	Male and Man	Disability assistance
