# Supplementary material for: Priorities for quality of life after traumatic brain injury
Source: PLoS One. 2024 Jul 5;19(7):e0306524. doi: 10.1371/journal.pone.0306524 (PMC11226113; doi:10.1371/journal.pone.0306524)
Supplement: S1 Appendix — (DOCX) [file pone.0306524.s001.docx]

**S1 Appendix**

**Focus Group Discussion Guide**

*Note: Questions & probes will be provided to all participants prior to focus group sessions.*

**Title:** Understanding Priorities of Quality of Life in People after Traumatic Brain Injury

**Discussion**

To start, let’s have everyone introduce ourselves. You can talk about when you had the traumatic brain injury, how long has it been since the brain injury.

Questions:

Q1. How would you define quality of life?

Q2. What do you think contributes to your quality of life after traumatic brain injury?

Q3. What activities provide value and quality in your everyday life?

Q4. How, if at all, is social connectedness or support important when determining your quality of life after traumatic brain injury?

Q5. How, if at all, is support (professional or personal) important when determining your quality of life after traumatic brain injury?

Q6. How, if at all, has your priorities for quality of life changed after traumatic brain injury?
