# Supplementary material for: Priorities for quality of life after traumatic brain injury
Source: PLoS One. 2024 Jul 5;19(7):e0306524. doi: 10.1371/journal.pone.0306524 (PMC11226113; doi:10.1371/journal.pone.0306524)
Supplement: S1 Table — (DOCX) [file pone.0306524.s002.docx]

Table 1: Factors that influence QOL (phase two)

| **Factors that Influence QOL** | **Number of respondents** |
| --- | --- |
| **Person factors** | |
| Social (e.g., relationships, support, roles, interactions) | 33 |
| Physical health | 21 |
| Recreation (e.g., hobbies and travel) | 15 |
| Well-balanced diet | 11 |
| Overall health | 6 |
| Financial capacity | 7 |
| Housing | 6 |
| Ability to perform daily tasks | 5 |
| Cognition | 3 |
| Adequate sleep | 5 |
| Feeling understood and respected | 3 |
| Spirituality | 2 |
| Self-awareness | 2 |
| Managing symptoms of TBI | 2 |
| Mental health | 4 |
| Education | 2 |
| Having basic needs met | 1 |
| Managing stress | 1 |
| Self-accepting injury | 1 |
| Having freedom | 1 |
| Taking medication | 1 |
| Having hope | 1 |
| **Activity-related factors** | |
| Employment capacity | 2 |
| **Environment factors** | |
| Accessible healthcare | 8 |
| Accessible transportation | 3 |
| Nature | 3 |
| Affordable services | 2 |

*NOTE*. Total *n* = 34. Participants outlined factors that influenced their QOL after TBI.
