# Supplementary material for: Priorities for quality of life after traumatic brain injury
Source: PLoS One. 2024 Jul 5;19(7):e0306524. doi: 10.1371/journal.pone.0306524 (PMC11226113; doi:10.1371/journal.pone.0306524)
Supplement: S2 Table — (DOCX) [file pone.0306524.s003.docx]

Table 2: Support after TBI (phase two)

| **Current Important Relationships Providing Support** | **Number of respondents** |
| --- | --- |
| Rehabilitation professionals | 23 |
| Physicians | 15 |
| Mental health support personnel | 9 |
| Community organizations | 9 |
| Informal supports (e.g., family, friends) | 6 |
| No support | 2 |
| Did not answer | 1 |
| **Current Community Support Received** | **Number of respondents** |
| Relationships with family or friends | 17 |
| Community organizations or services | 19 |
| Physicians | 14 |
| Rehabilitation professionals | 6 |
| No support | 5 |
| **Current support levels** | **Number of respondents** |
| Well supported | 18 |
| Somewhat supported | 2 |
| Not supported | 13 |
| Did not answer | 1 |

*NOTE*. Total *n* = 34. Participants outlined their support after TBI.
