# Supplementary material for: Priorities for quality of life after traumatic brain injury
Source: PLoS One. 2024 Jul 5;19(7):e0306524. doi: 10.1371/journal.pone.0306524 (PMC11226113; doi:10.1371/journal.pone.0306524)
Supplement: S3 Table — (DOCX) [file pone.0306524.s004.docx]

Table S3. Relevance of Priorities

| **Priority** | **Relevance** |
| --- | --- |
| Ensuring basic needs are met | highly relevant (n = 4) |
| Participating in everyday life | highly relevant (n = 4) |
| Trusting a circle of care | highly relevant (n = 4) |
| Finding meaning in relationships | highly relevant (n = 4) |
| Being seen and accepted | highly relevant (n = 3) moderately relevant (n = 1) |
| Achieving purpose and meaning | highly relevant (n = 3) moderately relevant (n = 1) |
| Giving back and advocating | highly relevant (n = 2) moderately relevant (n = 2) |

*NOTE*. Total *n* = 4. Expert participants rated relevance of the priorities for QOL after TBI.
